# Men’s reactions to receiving objective feedback on their weight, BMI and other health risk indicators

**DOI:** 10.1186/s12889-018-5179-1

**Published:** 2018-02-27

**Authors:** Craig Donnachie, Sally Wyke, Kate Hunt

**Affiliations:** 1 MRC/CSO Social and Public Health Sciences Unit, Institute of Health and Wellbeing, University of Glasgow, 200 Renfield Street, Glasgow, G2 3QB UK; 2 Institute of Health and Wellbeing, College of Social Science, University of Glasgow, 25-29 Bute Gardens, Glasgow, G12 8RS UK; 3 Institute for Social Marketing, University of Stirling, Stirling, FK9 4LA UK

**Keywords:** Health risk status, Behaviour change, Men’s health, Obesity, Weight loss intervention

## Abstract

**Background:**

Receiving information about one’s weight, Body Mass Index (BMI) and other indicators of health risk may prompt behaviour change. This study investigated men’s reactions to receiving information on indicators of health risk prior to taking part in a men-only weight management programme, Football Fans in Training (FFIT). It also investigated the extent to which the information was reported as influencing lifestyle change and having adverse consequences.

**Methods:**

We undertook a qualitative, semi-structured, telephone interview study with 28 men who took part in FFIT. We sought to interview approximately equal numbers of men who had and had not lost 5% or more of their pre-programme body weight by the end of the 12-week programme. Data were analysed thematically utilising principles of framework analysis.

**Results:**

Some men were apprehensive about receiving information which confirmed their overweight/obese status, particularly those less familiar with having similar information fed back to them. The professional football setting and the people present (including other men on the programme whom they perceived to be ‘like them’ and the fieldwork staff) were important factors in making the men feel comfortable in an otherwise potentially threatening situation. Men who achieved greater weight loss were more likely to report being motivated by this pre-programme feedback and to perceive themselves as responsible for their current weight and health status. However, for others the information only reaffirmed what they suspected about their relatively poor health status and was insufficient to prompt behaviour change.

**Conclusion:**

Undertaking measurements and receiving information on health risk indicators, such as weight or BMI, within the context of behaviour change programmes can enhance motivation for behaviour change when communicated in an empathic and non-stigmatising way, and therefore should be considered as an integral part of interventions. However, providing feedback on health risk may be insufficient to prompt behaviour change in some people and may be detrimental to those with poor body image and/or lacking personal agency to adopt lifestyle changes. It is therefore imperative that adequate support and opportunities are made available when information on weight and disease risk are fed back within research or other settings.

**Electronic supplementary material:**

The online version of this article (10.1186/s12889-018-5179-1) contains supplementary material, which is available to authorized users.

## Background

### Obesity and men’s reluctance to engage in weight loss initiatives

Increasing prevalence of obesity is a challenge for global public health [1], contributing to approximately three million deaths each year from obesity-related diseases [2, 3], although some dispute “conventional thinking about the unqualified dangers of obesity” given evidence that some people who are overweight and obese are not at increased risk for mortality [4, 5]. If recent projections endure, around 60% of men will be classified as clinically ‘obese’ in the United Kingdom (UK) by 2050 [6]. Scotland has the highest obesity levels among males in Europe, with around 70% of men classed as ‘overweight’ or ‘obese’ [7]. Moderate (5–10%) weight loss can produce clinical health benefits [8, 9]. However, men are under-represented in NHS and commercial weight management initiatives and in randomised trials of weight loss interventions and little is known about men’s experiences of weight and weight loss, particularly factors most salient in motivating men to lose weight [10].

Behavioural approaches can support weight loss in the short term, although weight is typically regained at a rate of approximately 1–2 kg/year [11], with a gradual return to baseline levels [12]. Hence, the long term efficacy of current public health approaches to overweight and obesity has been questioned [13]. Whilst Body Mass Index (BMI) and ‘excess’ weight are often taken to be useful indicators of future risk of ill-health, this is not universally accepted. Some dispute medicalised conceptions of ‘overweight’ and ‘obesity’, particularly the utility and legitimacy of the BMI (e.g. [14, 15]), and consider BMI to be a crude proxy for adiposity and ill-health (e.g. [16, 17]). Moreover, it has been argued that medicalisation of ‘excess’ body weight within current public health approaches can compound weight bias and stigma [18–20] and marginalise individuals [21]. Consequently, some have advocated for a less weight-centric approach to weight management, such as the Health at Every Size (HAES) paradigm [18, 22] which advocates promotion of health and wellbeing through increased physical activity and healthful eating [22]. However, the majority of studies addressing this approach again consist disproportionately of (white) women who have “a history of binge eating or chronic dieting” [23]. Additionally, some argue that moving away entirely from weight loss as a public health focus may further marginalise individuals who are actively seeking support for weight management, particularly those for whom a modest reduction in weight could yield substantial health benefits, despite their BMI remaining in the overweight or obese range [23, 24].

In recent years research has considered interrelationships between men’s health behaviours and practices and constructions of masculinity (e.g. [25]). It has been suggested that cultural constructions of masculinity may undermine men’s attempts to lose weight [26, 27]. Men have been said to be more likely to: perceive dieting as ‘feminised’ behaviours (e.g. [28]); control their weight via exercise alone [29]; contest biomedical definitions of ‘obesity’ (e.g. [16]); be satisfied with their body shape/size when overweight, desiring not to be perceived as being ‘too thin’ (e.g. [30]); and underestimate their susceptibility to obesity-related diseases [31].

Research on young men’s health behaviours [32] has suggested that competence in traditionally masculine health-related domains can accrue masculine ‘credit’ or ‘capital’ which can then be drawn upon or ‘traded’, in order to legitimise other behaviours or practices which are perceived as conflicting with cultural ideals of masculinity. These conceptualisations of masculinities suggest particular utility in developing public health initiatives for men which are designed to work with, instead of against, prevailing notions of masculinity. Professional sports settings have been seen as a promising ‘masculine’ context for engaging men at ‘high risk’ of ill-health (e.g. [33, 34]). For example, recent evidence showed how a weight management programme delivered via professional football clubs (Football Fans in Training, FFIT), attracted, engaged and enabled men to lose weight, increase their physical activity and adopt a healthier diet [27, 35].

### Feedback on weight and other health risk indicators

Numerous studies have investigated the ways in which people respond to information about their health risk, specifically personalised clinical or biomarker feedback (e.g. [36, 37]), and offering information about health markers may challenge perceptions of invulnerability [38]. Evidence also suggests that men may experience a shift in their attitudes towards health and health behaviours as they experience the effects of ageing [39–41]. Hence, receiving personally relevant information on potential indicators of health risk may have particular salience for men in early middle age and beyond. For instance, a small-scale evaluation of a weight management programme delivered through a men’s health clinic [42], reported that, in this circumstance, being told sensitively by a healthcare professional who had measured their height and weight as part of a ‘men’s health check’ that their BMI fell above the threshold for ‘obesity’, was a powerful prompt for deciding to lose weight [42]. These findings are echoed in a recent comprehensive review of qualitative evidence demonstrating knowing whether one’s BMI exceeds the threshold for ‘obesity’ can enhance men’s motivation to engage in weight loss efforts [10].

However, weight stigma is prevalent throughout Western culture and it is well known that terms used to describe being overweight can carry moral overtones [19, 43] or enhance experiences of stigma [44–46]. There is also evidence indicating that weight stigma is associated with a range of adverse physical, psychological and behavioural outcomes (e.g. [47]), and Puhl and colleagues argue that obesity-related public health messages perceived as stigmatising, shaming or blaming may hinder motivation for behaviour change [48]. In a qualitative study [49] investigating reactions to receiving personalised feedback on objectively-measured weight, BMI and other health risk indicators in participants in a longitudinal cohort study, some overweight participants used predominantly negative language to describe their reactions to the feedback, including feelings of distress, powerlessness and anxiety about their future health. However, other participants discussed increased motivation or assimilating lifestyle changes in response to this feedback. The authors concluded that, for some, the motivational aspects of weight-related feedback may outweigh the potential adverse consequences [49], and that some people appreciate a more direct approach when discussing their weight status with healthcare professionals/in a medical context, when use of weight-related terms such as ‘clinically obese’ may be perceived as motivating rather than stigmatising [50]. For instance, recent findings demonstrate the effectiveness of a screening and brief intervention delivered by primary care physicians to encourage patients to lose weight [51].

It is thus recognised that providing individualised feedback to participants within research studies may constitute an intervention in itself [52]. Findings from the randomised controlled trial (RCT) of the FFIT programme showed that 11% of men in the waiting list comparison group weighed at least 5% less than their baseline weight at 12-month follow-up, i.e. *prior* to being offered the 12-week FFIT programme. The authors suggested that receiving individualised information on their weight, BMI, waist and blood pressure measurements, at baseline measurements sessions, in combination with an advice booklet and some information about the programme, may have been enough to motivate some men to lose weight [35]. However, despite evidence that knowing one’s ‘obesity’ status can provide a potent source of motivation [10], the ways in which men who are overweight or obese interpret and/or react to information on their weight, BMI or other possible health risk indicators in research and other settings is not well understood and it is conceivable that providing feedback on weight status has the potential for adverse consequences, such as feelings of distress, shame or discomfort. Given the negative consequences of weight discrimination and stigma, it is vital to optimise communication of weight-related information within research and/or behavioural interventions and to debate the ethical ramifications of providing feedback. The aim of this qualitative study is thus to understand: men’s reactions to receiving information on objectively measured health risk indicators prior to taking part in a men-only weight loss and healthy living programme (FFIT); and their capacity to influence lifestyle change or have adverse consequences.

## Methods

### Research context for the study: Football fans in training (FFIT)

FFIT is a weight loss and healthy living programme, delivered free of charge over 12 weeks at professional Football Clubs by community coaches to men classed as overweight or obese (BMI > 28 kg/m^2^) aged 35–65 [53]. FFIT was developed to work alongside common constructions of masculinity, in recognition of health behaviours as an important means of ‘doing’ gender [26]. Hence, FFIT was designed to appeal to men in: context, content and style of delivery, drawing on evidence of what is most effective for weight loss [54], including a range of behaviour change techniques shown to be effective in physical activity and healthy eating interventions [55–57]. The FFIT programme is positioned as an opportunity to support men to improve their lifestyles and feel better through increased fitness and losing weight through a group-based programme alongside other men with an interest in football.

All participants in this study took part in deliveries of FFIT commencing in January/February 2012. They undertook a suite of objective measurements at the club stadium immediately before commencing FFIT (‘baseline’) and 12-weeks later (‘post-programme‘). Measurements were conducted by fieldwork staff trained to the same standard protocols as used in the FFIT RCT [27, 35]. Weight (kg) was assessed using electronic scales (Tanita HD 352). Height (m) was recorded using a portable stadiometer (Seca Leicester). Waist circumference was measured twice with a 200 (cm) tape. A third measurement was made if the first two varied by ≥5 (mm) and mean waist size was calculated. Resting blood pressure was recorded using digital monitors (Omron HEM-705CP) which were calibrated before fieldwork commenced.

Information on some of these physical measurements was fed back to men by the fieldwork staff at the baseline measurements; fieldworkers were trained to explain what each measurement meant. Men with elevated blood pressure readings were given a letter documenting these readings and were encouraged to consult their GP. Fieldworkers were trained to use BMI wheels as a tool to demonstrate visually where participants BMI fell within the overweight or obese range, using conventional cut-offs [58]. Training on the appropriate wording to use was based on prior research demonstrating the importance of using language that minimizes offence or distress when discussing information on weight and health risk status, while also ensuring that possible health implications of a modest (5–10%) reduction in weight are clearly communicated [50].

### Design

We invited men taking part in ‘non-trial’ deliveries of FFIT at four Scottish professional football clubs commencing in February 2012 to participate in a qualitative, semi-structured, telephone interview study [59]. This study was part of a UK MRC funded PhD project, which aimed to understand men’s experiences of self-monitoring their physical activity both in the initial stages and after doing the FFIT programme, receiving objective feedback on objectively measured health risk indicators before the programme, and their objectively measured activity levels before and after doing the FFIT programme see [59, 60]. One aim of the interviews was to understand men’s experiences of receiving feedback on indicators of health risk, specifically information on the objective baseline measurements described above. All respondents had received an information sheet about the study, had met the researcher at the baseline measurement session and knew they might be invited to take part. We sought to purposively sample equal numbers who had and had not lost at least 5% of their baseline weight during their participation in the 12-week programme, to explore whether there were differences in the accounts of these two groups. Of the 28 men who agreed to be interviewed, 14 had lost 5% or more of their baseline weight at post-programme measurements, whereas 12 had not; weight outcomes were missing for two men (see Table 1). All participants are made aware at the beginning of the FFIT programme what a 5% weight loss target would be for them.

**Table 1:** Participant characteristics, ordered alphabetically by pseudonym

Pseudonym	BMI category (baseline)	Age	Marital status	SIMD	BMI (baseline)	Waist circumference (cm) (baseline)	Achieved at least 5% weight loss 12-weeks	% weight loss 12-weeks	Club ID
Alan	Obesity I	44	Single	5	31.3	104.0	No	2.06	02
Alex	Obesity III	42	Cohabiting	1	41.9	137.3	Yes	5.94	01
Andrew	Obesity II	41	Married	1	36.5	124.0	No	3.82	02
Ben	Obesity I	36	Separated	3	34.4	115.1	No	n/a^b^	04
Billy	Obesity II	52	Married	5	38.9	129.8	Yes	7.5	03
Calum	Obesity II	38	Divorced	1	39.7	132.8	Yes	5.17	01
Chris	Overweight	58	Married	5	28.5	104.5	Yes	8.8	02
Dan	Overweight	58	Married	5	26.6	105.3	No	n/a^b^	02
David	Overweight	49	Separated	3	29.1	99.3	Yes	5.74	04
Donald	Obesity III	49	Married	2	46.0	129.4	No	n/a^b^	04
Frank	Obesity II	49	Married	1	35.7	112.6	Yes	9.67	01
Gary	Obesity I	50	Married	5	32.0	106.1	No	4.32	02
George	Obesity II	56	Married	5	35.2	112.2	Missing^a^	Missing^a^	03
Gordon	Obesity I	40	Divorced	1	34.6	115.5	No	1.87	04
Grant	Obesity III	58	Married	1	42.0	127.2	No	2.09	04
James	Obesity I	42	Cohabiting	2	30.5	114.0	No	3.5	04
Jamie	Obesity I	36	Married	3	31.3	114.8	Yes	6.07	02
Jeffrey	Obesity I	53	Cohabiting	2	33.1	118.3	Yes	9.78	04
Jonathan	Obesity III	47	Married	2	43.0	135.9	Yes	15.99	03
Kevin	Obesity I	47	Cohabiting	3	32.0	105.1	No	n/a^b^	02
Martin	Obesity III	34	Married	2	42.8	143.1	Yes	10.31	02
Matthew	Obesity III	47	Married	3	40.4	124.2	Missing^a^	Missing^a^	03
Michael	Overweight	55	Married	2	27.1	97.1	Yes	7.58	01
Ross	Obesity II	52	Married	2	38.4	131.3	No	1.92	02
Ryan	Obesity III	54	Married	4	42.4	132.2	Yes	11.52	03
Steven	Obesity II	33	Married	4	36.9	120.8	Yes	5.43	01
Thomas	Overweight	37	Separated	3	29.1	108.2	No	1.65	02
Tim	Obesity I	33	Cohabiting	5	31.7	110.8	Yes	6.86	02

^a^Missing: data missing, ^b^n/a (non-applicable): did not achieve weight loss, *SIMD* Indicator of level of affluence/deprivation of areas of residence using Scottish Index of Multiple Deprivation (Quintiles), 5 = lowest quintile of deprivation

Data were analysed thematically utilising the framework approach [61, 62]. The framework method is not positioned alongside a specific epistemological, theoretical or philosophical approach, so offers a flexible tool for qualitative researchers that can be adapted for use in inductive or deductive analysis or a combination of both [63]. The framework approach starts deductively from the aims and objectives of the study, with the overall findings being grounded and inductive in the original accounts of those studied [64]. A defining feature of the framework method involves the matrix output, whereby rows (cases), columns (codes) and ‘cells’ of summarised data provide a rigorous approach for the management and analysis of qualitative data [63]. This procedure enables scientific rigour to be maintained due to the methodical and systematic nature of this approach [65]. Thus, the framework approach enabled us to address our overarching aim of understanding men’s reactions and responses to receiving information on indicators of health risk with interpretations of the data generated grounded in the men’s own accounts. Ethical approval was granted by the University of Glasgow, College of Social Sciences Research Ethics Committee (CSS201020106).

### Data collection

The first author and primary analyst (CD) conducted the semi-structured telephone interviews, seated in a quiet, private university room to ensure optimal data collection and confidentiality. CD had previously met all men twice (at baseline and post-programme measures), establishing a personal connection that was vital in building rapport prior to the telephone interviews. CD was as a member of field staff during initial baseline assessments at clubs (prior to randomisation) and attended baseline and follow-up sessions at four clubs in ‘non-trial’ deliveries of FFIT in February 2012 as part of the study (as described above [60]). At baseline assessments in January/February 2012, he did not undertake any of the measures reported here, but met men at the end of the measurement session to ask if they would be willing to wear an activity monitor and to ask in principle if they would be willing to take part in this additional research. Interviews were conducted between September 2012 and February 2013, and most lasted 60 to 90 min. The interview guide included a range of topics, including men’s experiences of receiving objective feedback on health risk indicators during the measurement sessions. For example, “What was it like getting the feedback?” and “Did this feedback make you think differently about yourself?” (see Additional file 1; for full version see [60]).

### Data management and analysis

Interviews were digitally recorded with participants’ consent onto an internal server to ensure security and optimise recording quality. CD checked each transcript against the original recording for accuracy. Respondents were given pseudonyms and clubs were allocated identification numbers to ensure de-identification. Nvivo software (QSR International Pty Ltd. Version 10, 2012) was used to facilitate data storage and retrieval.

In accordance with the framework method [61, 62], each transcript was read several times by CD prior to formal analysis. During this process of familiarisation, initial thoughts were noted focusing mainly on what respondents said about receiving feedback during the baseline measurement sessions; SW and KH read a sample of transcripts to verify key themes. CD then initially coded to agreed broad headings regarding what men said about being told their objective measurements at baseline. The following headings emerged from the data and were corroborated through discussion of the coded data with co-authors (SW and KH): men’s perceptions of health risk (in response to feedback); the influence of other people and the research context on measurement and provision of feedback; emotional responses; the objective baseline feedback as a benchmark; the information as confirmation of perceived overweight/health risk status; and feedback as a prompt for behaviour change. Outputs from these broad codes were read and discussed by all authors. Once the transcripts were coded to these themes, the content within each theme was charted and summarised into framework matrices by CD. This enabled us to visually interrogate data across each framework as well as referring back to the original transcripts. During this phase of the analytical process, we were able to interpret the data further, inferring higher level concepts and overarching themes. The four main themes that emerged from our analyses were: *Men’s anticipation of being measured prior to undertaking FFIT; Men’s experiences of being measured at baseline; Men’s experiences of receiving information on objectively measured indicators of health risk; and Impact of receiving information on objectively measured indicators of health risk.* To enhance rigour and trustworthiness, transcripts and thematic outputs were independently read and verified by co-authors (KH and SW) to allow detailed discussion of the data, coding and interpretation, at each stage of the analytical process.

The ‘One Sheet of Paper’ method [66] was employed at this stage of analysis to add further rigour and as a systematic check that all perspectives were represented in the findings. This approach requires close inspection of data coded to each theme and recording, under distinct headings, all examples of issues arising in the data, noting respondent details next to each extract. It allows the identification of the range of experiences under each main theme and any unexpected findings or ‘deviant cases’. Systematic comparisons of the accounts of men who had and had not achieved at least 5% weight loss post-programme were conducted, across the four consolidated themes, to identify any differences in language and responses used. The men were interviewed retrospectively, after they had participated in FFIT, and their recollections may have been influenced by their success (or otherwise) on the programme. The study adhered to the Consolidated Criteria for Reporting Qualitative Research guidelines [67]. Men were offered a £20 club shop voucher to thank them for doing the interview.

## Results

Respondents’ baseline characteristics are provided in Table 1. Their mean age was 46.1 years (sd 8.1), mean waist circumference was 118.2 cm (sd 12.5) and mean BMI was 35.4 kg/m^2^ (sd 5.4). Using conventional cut-offs, five men were classed as ‘overweight’ (BMI 25–29.99), nine as ‘mildly obese’ (BMI 30–34.99), seven as ‘moderately obese’ (BMI 35–39.99) and seven as ‘extremely obese’ (BMI ≥ 40). These characteristics demonstrate that respondents were similar to participants in RCT and pilot research deliveries of FFIT [33, 35].

As described above, while attending baseline measurement sessions men underwent a battery of objective physical measurements (including body weight, height, waist circumference, BMI, blood pressure and respiratory function). They were invited over to each of the measurement stations individually where the information on each of the measures was recorded in their participant questionnaires and fed back to them verbally by the field staff; in between these measurements men spent time filling in a self-completion questionnaire. During the interviews the men spoke predominantly about their experience of the weight, waist, BMI and blood pressure assessments. These measurements are therefore the ones which are referred to throughout the findings presented (Table 1).

### Men’s anticipation of being measured prior to undertaking FFIT

Men who had experience of being measured in other contexts, such as work (e.g. routine health assessments) or as a consequence of long-standing medical conditions (e.g. high blood pressure or diabetes), talked about it ‘not being a problem’ or being ‘routine’:

*Not a problem […] I used to get an annual medical, physical, so a lot of the tests were kinda similar [...] it was absolutely no problem, you know, it was like waist, lung capacity, blood pressure, all that sort of stuff so it’s fairly standard. (Gary).*

*I mean it was just like going for a proper medical examination tae [to] see how fit and what kinda shape you were in type of thing, aye. I knew before I went that, I mean I’m on tablets for high blood pressure anyway, so I knew that that was gonnae be okay, cos [because] that was controlled by the tablets. (Ross).*

Others used language which suggested they had been anxious about attending the initial measurement sessions. For example, Michael said “I forced myself to go to the programme and actually get the measurements.” A few men used emotive language to articulate how they felt in anticipation of being measured, indicating shame, fear or anxieties about their body:

*I was really nervous getting all the measurements done, cos when I started, yeah I was so nervous because I was so ashamed of my body, and I wasn’t really wanted [wanting] any of that done basically, but I knew I had to get it done. (Calum).*

Some men were apprehensive about receiving information which might confirm that they had an elevated risk of future ill-health. For example, Dan reported feeling concerned about his blood pressure and cardiovascular system: “Obviously in the back of your mind there is a sorta [sort of] worry if you want, about your blood pressure, your cardiac system, everything, you know.”

Thus whilst some felt very anxious about having physical measurements done, men who had experience of regular medical checks were less likely to describe concerns about attending the measurement session.

### Men’s experiences of being measured at baseline

Once the men had gone through the sometimes challenging process of deciding to attend the initial measurement session, the majority described the process of being measured as a relatively ‘*positive*’ experience. For instance Calum, who had been very apprehensive about going, said: “When we started [...] I was like ‘oh I’m dreadin’ this’ but I done it and I quite enjoyed it actually, to be truthfully honest.” Indeed, many welcomed the opportunity to be measured. For example, Gordon remarked “I’ve never actually been measured to that extent before”. A few described receiving the information as an important ‘*benchmark*’ against which they could compare themselves as they progressed through the 12-week programme and beyond:

*It was very positive, especially when I first started to find out exactly how much I weighed because I didn’t know at that time and then obviously keeping a tab on how much I’d lost over the duration of the course. (Alex).*

Some men explicitly highlighted perceived barriers to accessing these kinds of physical measurements. For example, James stated: “that's quite difficult accessing, or getting your GP to even do that. […] last time I contacted them, they said it would cost £108 to get a basic health check done.” Similarly, Thomas said: “I always wanted to know […] in terms of my target weight. But it’s not something you would go to the doctor for, just to get weighed”.

Several factors were important in ensuring that men felt comfortable in a situation they might otherwise have perceived as threatening. Some highlighted the importance of the setting – and described being measured *within the professional football club* as being ‘*inspiring*’ and ‘*motivating*’:

*I found it quite invigorating and liberating and quite motivating actually to go there and it was done [...] in a very relaxed and [in]formal atmosphere and very positive, so no I thought it was a, a very positive experience, one that provided a good motivation for, for making changes. [...] I did it at the ground and there was other people there in the programme and I think that in itself was motivating, that you’re part of a group all with the same purpose. (Ben).*

But, this quote highlights too that *the people*, including other men in the group, club coaches and the fieldwork staff, were also important in creating a supportive environment for undergoing the physical measurements. A few described the process as being a humorous or ‘funny’ experience:

*Yeah, well, initially when we first started, there was quite a few of us there and were all having a kinda laugh and joke about it. And then everybody got measured you know, and obviously everybody, well, most of the people were a bit overweight. It was a bit of ae [of] a laugh and a joke. (David).*

Most men emphasised the importance of being made to feel ‘comfortable’ or ‘at ease’ during the measurement process and described the fieldwork staff, who took the measurements, as ‘friendly’, ‘professional’ and ‘supportive’:

*I think the actual [...] process [measurement] [...] was done in a way that wasn’t embarrassing or threatening [...] the people who were taking the various recordings on my weight, my height,* etc. *[...] all of those kind of individual aspects of the recordings, did it in a way that you know made it as easy as possible or approachable, friendly, kind of I suppose tried to detract from any potential stress associated with that. (Frank).*

Frank’s comments highlight an underlying recognition that the measurements could have been ‘embarrassing’ or ‘threatening’ without the reassuring approach and demeanour of the fieldworkers. The men reported how they felt the fieldworkers had invested effort into helping them by showing genuine interest and care. For example, Jonathan spoke about how he felt “a certain level of friendship [...] and people actually cared about you as an individual rather than you were just a number attending a course”.

Most men valued being told the reasons for each of the measurements. For instance, Martin reported how the information he received provided him with valuable information:

*Speaking to them [fieldworkers] more in depth, ‘cause the doctor just used to say, ken [you know], right, “Take these [...] tablets and then that’ll be fine!”. Ken, they really never gave much of a guideline tae what I was and what I was doing to myself with that. I cannae mind [* *can’t* *remember] the woman’s name that [was] daen’ [doing] my blood pressure. She did, she definitely laid it on the line, you know, what it was that was goin’ tae happen [...] I thought about it more when I was getting them done there, ken, rather than just taking tablets all the time.*

Therefore, the analysis showed that it was possible to undertake measurements of weight and body size in an unthreatening and non-stigmatising way and that some men appeared more cognisant of the meaning of their health-related measurements and how these specifically related to their individual lifestyles and behaviours in response to the process of measurement and feedback.

### Emotional responses to information on health risk indicators

Some men used emotive language to express how receiving this information made them feel. For instance, several men described feeling shocked when they found out how much they weighed:

*I knew, myself, I was getting big but when I realised I was over eighteen stone [114.3 kg], I thought that was a bit ae [of] a shock and it wasnae very good, so I knew something had to get done pretty drastically. (Jamie).*

Some men reported feeling particularly shocked when they were told their BMI classification, especially those who were told that their BMI placed them in the ‘clinically obese’ category:

*Well that’s, that was the first time I’d ever seen my BMI. I’d never, I’d heard about it but I’d never actually knew what it was, what it meant, [...] to find out that I’m first called ‘clinically obese’ was a bit of a shock. I knew I was overweight but I didn’t think it was ‘clinically obese’ so that was a bit of a kinda kick in the teeth. (Gordon).*

A few described other emotional responses. For example, Michael felt ‘annoyed’ at himself:

*I was probably more annoyed that I’d let myself get to that state. [...] I was always fit, so for me to get to that state [...] I would probably have said the overriding emotion in me was annoyance in allowing myself to get to that state.*

Ryan described feeling ‘disgusted’ when he was told he was clinically obese on the basis of his weight and height:

*I knew before I started the, the, the course I was what you would call was ‘clinically obese’ because of my body ma— I, I realised that. Different people perceive it in different ways. I felt a bit ... phew ... how can I put it ... I felt not shocked, I felt disgusted wi’ myself when they said “you’re o ... o ... [obese]”– I knew I was obviously obese.*

In contrast, some men seemed less affected by receiving feedback on their measurements. For example, Grant described receiving information about his baseline measurements as having minimal impact emotionally or otherwise:

*None of the results bothered me – or was surprised by them or anything like that. Taking the measurements – probably just one of these things that I had tae [to] do to get through to get tae the ... the programme. And – and nothing really fazed me I didn’t really have any problems with it, you know? […] I wasn’t really fazed again by any of these, eh, measurements and sizes […] they don’t bother me. I’d love tae be – eh – you know - a thin guy but the fact that I might be 54 or 52 round the chest, I don’t really know what I am noo [now], whatever it was, it doesnae bother me. It bothers me the fact I cannae go and buy […] you know – Hugo Boss or whatever it is […] other than that, it didn’t bother me.*

Grant was also one of three men who openly questioned the validity of their BMI as an accurate indicator of obesity or health risk status. He said:

*Well BMI I’ve gottae say is a hundred years old. The BMI, I don’t – I don’t take much significance on BMI, right? BMI, tae me, the – the whole of the English rugby team who won the World Cup were all obese due to this BMI. BMI doesn’t take any consider[ation] of any muscle content or – or – you know – they’re all overweight because of their muscles and, eh, I don’t really – I’m no’ really bothered about BMI.*

### Impact of receiving information on indicators of health risk

There were a range of responses regarding what the men said about utilising the information and the effect that it had on them. Some men said the information confirmed what they thought or suspected about their health risk status, which, in turn, reinforced their reasons for wanting to attend the programme and/or embrace lifestyle changes. For example, Tim mentioned: “Hearing things like your BMI and [...] what band that's within [...] just reinforced to me that I could really do with making a change.”

However, some explicitly said that receiving the information was not sufficient in itself to motivate subsequent behaviour change. For them, various components of the FFIT programme were perceived as more important in facilitating behaviour change:

*I don’t think the actual measurements made me think I need tae make the changes, I think it was more to do wi’ [with] [...] going along to the programme and the support of the coaching staff plus also the other participants, you know. (James).*

In contrast, other men said the information encouraged and motivated them to make changes and reported greater perceptions of vulnerability to ill-health as a result of receiving information on their measurements:

*I was relatively near retiral age as a nurse and I kind of thought that the last thing I wanted to see was myself [...] having [...] a stroke or some kind of physical, serious physical problem because of my weight. And at that point I kind of thought “Well this information confirms that that’s more likely than less likely and I needed to do something about it”. (Frank).*

These men were more likely to use language such as ‘my fault’ or ‘self-inflicted’ suggesting they were more likely to attribute their weight/health risk status to their own behavioural choices, which invoked a desire to implement lifestyle changes:

*I’m trying to think how to put it, how I felt. “Aye it’s time now to do something about it before it’s too late,” […] I started to think about my family and different things like that as well that, “It’s time I really did something about the problems.” Because it’s more or less self-inflicted – my weight issue and things. (Jonathan).*

These men also used language such as ‘drastically’ or ‘now or never’ to describe their instant reactions to the information and their desire to take immediate action:

*The truth was there, it was just I was very overweight and seeing it all written down in front of me, how big I was, how unfit I was, how unhealthy I was, made me realise I’ve had tae dae* *[to do]* *it. It was basically a now or never and I’m glad I choose the, the now if you know what I mean (Martin).*

Detailed and rigorous comparisons during the later stages of analysis revealed that the men, who described being motivated to lose weight and adopt lifestyle changes in response to the information, were more likely to be those who had lost more than 5% of their baseline weight by the end of the 12-week programme as demonstrated by their weight loss outcomes.

## Discussion

The aim of this analysis was to understand men’s reactions and responses to receiving information on objectively assessed indicators of health risk at baseline assessment prior to commencing a 12-week men-only weight loss programme. The findings illustrate that a few men were apprehensive about receiving information pre-programme which confirmed their overweight status and/or elevated health risk, especially those who were unfamiliar with having similar assessments performed previously. The findings reveal that the professional football club setting and the people, including other men who they saw as being like them and the fieldwork staff, were important factors in making the men feel comfortable. Men who were most successful in losing weight during the programme were more likely to explicitly state that they had felt motivated to implement lifestyle changes in response to the information provided at baseline. They were also more likely to attribute some responsibility for their own weight/health status and express a desire to make immediate lifestyle changes. However, for some men the information merely reaffirmed what they already suspected about their weight/health risk status and was not sufficient to motivate behaviour change. Overall, these findings contribute to a growing evidence base indicating that awareness of whether and to what extent one’s BMI exceeds the threshold for ‘obesity’ may enhance men’s motivation to take action [10].

Some men said they had felt anxious about attending the baseline measurements, some expressed embarrassment or shame and used language to suggest they had poor body image, and still others reported feeling concerned about receiving information which might confirm an increased risk of ill-health. These findings echo previous research with men from other deliveries of FFIT [27], specifically feelings of nervousness or embarrassment in anticipation of having weight-related assessments. The findings also resonate with recent evidence indicating that in addition to health concerns, body image and appearance concerns are salient issues for middle-aged, overweight men [68]. Taken together these findings suggest that overweight men may evade encounters which increase feelings of anxiety about their bodies and/or undermine their perceived capacity to effectively implement lifestyle changes, especially among those unfamiliar with similar forms of assessment. These findings are thus consistent with previous evidence indicating that specific weight-related terms may be regarded as stigmatising [44–46]. However, they also demonstrate that it is possible to undertake these measurements and convey the results in ways which reduce the extent to which they are perceived as threatening or stigmatising.

These findings can also be interpreted through the lens of Self-Determination Theory SDT; [69, 70], a prominent theory of motivation and health behaviour change. The SDT framework outlines three basic psychological needs as being fundamental for health, motivation and optimal functioning, namely *autonomy, competence* and *relatedness*. In line with SDT, feelings of shame or embarrassment are likely to impede one’s feelings of *competence*, a crucial antecedent for motivation and wellbeing. According to the SDT framework, competence is the need to feel optimally challenged and able to interact effectively in one’s environment. Moreover, within SDT, *autonomy* is defined as the need to feel volitional and a sense of authorship of one’s actions, rather than externally pressured or coerced, and is theorised as vital for optimal motivation and wellbeing. Therefore, men who feel ashamed of their bodies and have low self-worth may feel ill-equipped to effectively alter their lifestyles. Similarly, men concerned about their health status may lack the capacity or confidence to implement behavioural changes prior to attending baseline measurements. Further, it has been suggested that negative stereotypes and social ideals, especially relating to body image, may hinder feelings of autonomy [71]. Some have argued that dominant sociocultural ideals for accepted and desired masculine bodies in Western countries have shifted in recent decades (e.g. [72]) with men becoming more concerned with body image (e.g. [73]). Thus, some men might perceive themselves as falling short of current idealised standards in relation to their body image and experience increased feelings of body dissatisfaction [74]. Consequently, some men may feel external pressure to modify their bodies and experience decreased feelings of autonomy.

These findings demonstrate the ways in which the setting (i.e. professional football club) and the people within in it, including the fieldwork staff and other men ‘like them’ (i.e. similar body size, men interested in football), were important inter-related factors that ensured they felt comfortable and relaxed during the baseline measurements. Men described the professional football club setting as ‘*inspiring*’ and ‘*motivating*’, and the fieldworkers were viewed as professional and having invested time into helping them by showing genuine interest and care. Hence, the men’s descriptions are consistent with what the SDT framework defines as *relatedness,* i.e. the need to feel understood, respected, and cared for by others which, alongside autonomy and competence, is crucial for wellbeing and functioning.

Objective feedback that was recorded, and written down ‘in black and white’ by someone else, was viewed as being undeniable; some described this as being important in their resolve or readiness to take responsibility for their health. These findings resonate with previous suggestions that some men prefer more direct and result-oriented styles of communication (e.g. [75]). Receiving feedback on their weight status, and in particular being told in a sensitive way that, from a medical perspective their height and weight placed them above the threshold for being ‘clinically obese’, was an important motivator for several men and reconfirmed their reasons for enrolling on the programme. Some said they ‘knew’ they were overweight but had not realised the *extent* to which they were, supporting previous research demonstrating that men may only experience body dissatisfaction once they perceive themselves as obese [76]. However, in this study, some men were critical about some of the information they received on their physical measurements. Specifically, three men questioned or rejected the validity of BMI as an indicator of health risk. These findings are consistent with prior work indicating that men are more likely to refute the BMI as an indicator of unhealthy weight status [50] and may seek a body weight incongruent with biomedical definitions of a ‘normal’ or ‘healthy’ weight (e.g. [77]). They also resonate with literature querying the utility of the BMI as an indicator of ill-health (e.g. [16, 17]). However, despite some limitations others have advocated that the BMI is an acceptable metric for assessing excess adiposity [78]. Nevertheless, it is important to consider some of the limitations of relying solely on the BMI as a marker of individual health, particularly among specific populations (e.g. [79]).

For some men, information on their weight and other health risk indicators (e.g. blood pressure) were powerful motivators for action and were said to increase their resolve to implement behaviour change. In line with several theories of behaviour change (e.g. [80]) some men discussed weighing up their perceived likelihood of experiencing adverse health consequences in response to the information which, in turn, prompted them to take action. The findings are also consistent with theoretical notions of health behaviour change which posit that increased risk awareness (alongside outcome expectancies and perceived self-efficacy) are salient factors in the motivation phase of behaviour change [81, 82]. They are also consistent with research indicating that medical triggers or events can serve as teachable moments or motivators for weight loss and behaviour change (e.g. [83]). They also support previous research demonstrating that feedback on weight status was important in motivating men to participate in a weight management programme [42] and congruent with a recent review of the qualitative evidence in this area [10].

### Implications

The finding that some men experienced receipt of weight and health risk information as an important motivator has implications for future design of intervention studies. The findings also suggest that it is important, when inviting men to be measured as part of research studies or behavioural interventions, to address underlying fears about receiving information on health risk indicators, particularly for people who have limited experience of these kinds of measurements. They also demonstrate the importance of ensuring supportive contexts are promoted within weight management or health screening programmes, particularly where personal and sensitive information is communicated to individuals. Additionally, these findings indicate that receiving information on health risk indicators may have unintended consequences for participants within the context of weight management/behaviour change intervention programmes. Furthermore, the emotive language used by some men in this study to illustrate how receiving the information made them feel demonstrates the importance of ensuring sensitivity when discussing weight and/or health-related issues, even when presented in a non-stigmatising way. Puhl and colleagues reported that no matter what language or terms are used to describe excess body weight, they are likely to invoke an emotional response for the majority of individuals [45]. Therefore, it is important that sensitive information on weight, health or disease risk be made available or communicated to individuals judiciously in an empathetic and non-judgemental way within research or other settings [49]. Further, without adequate, accessible support, instruction or skills to enable lifestyle change, such information could negatively impact motivation and wellbeing.

### Limitations

The study had some limitations that are important to note. These men’s accounts were retrospective; men were asked about their responses to being measured at baseline after they had completed the FFIT programme. Hence, some accounts may have been subject to distortion or recall bias and influenced by subsequent success (or otherwise) on the programme. Furthermore, CD (first author) had previous contact with the men during baseline and post-programme measurement sessions. Whilst we believe that developing good rapport with the men was essential in gaining rich insight into their experiences during the telephone interviews, it is possible that some men may have felt reluctant to provide more negative perspectives despite being encouraged to express their opinions freely at all times. Finally, it is important to note that all the men who took part in this study had already taken the initiative to respond to programme recruitment materials which had branded FFIT as an opportunity to take part in a group-based programme which could support men to lose weight in the context of getting fitter and more active (recruitment fliers at the time used phrases such as: “Get fit. Shed a few pounds. Become more active at your local Scottish Premier League Club”; “Do you want to lose weight, and get fitter and healthier? Get involved with [name of professional football club] FFIT”).

## Conclusions

These findings show how; receiving objective feedback on potential health risk indicators, such as weight, BMI and blood pressure, can enhance men’s motivation to improve their health and embrace behaviour change, within the context of a gender-sensitised weight loss intervention; and that the context and manner in which such information is conveyed is crucial. Men who perceived themselves at greater risk of ill-health in response to the information demonstrated greater resolve and commitment in making lifestyle changes, as demonstrated by their 12-week weight loss outcomes. However, for others the information was not sufficient to prompt behaviour change. The findings also show that without adequate, accessible support, providing health risk information may undermine feelings of competence/agency, especially for those with poor body image/self-esteem or less experience of receiving similar kinds of information. It is therefore imperative that adequate resources and opportunities are made available to facilitate and encourage lifestyle changes when such information is fed back to individuals within research settings. These findings also demonstrate the importance of translating sensitive health and/or weight-related information to men, within environments that are consistent with their identities.

Future research investigating the longer term implications of providing feedback to men taking part in weight management and other research studies would be beneficial. Recent evidence suggests that women may appraise certain weight-related terms as less desirable than men, possibly because of their greater exposure to weight stigma [46]. Hence, further research examining the ways in which objective feedback on weight status and other health indicators are experienced by women would be enlightening, especially within research and other settings. Finally, future research examining the potential influence of receiving feedback (i.e. in relation to weight and other health risk factors) on motivation to perform health behaviours would be of benefit, particularly considering the myriad improvements behaviours such as physical activity and healthier eating habits can have on reducing various health risk markers, independently of weight loss.

## Additional file

Additional file 1: Interview Topic Guide. (DOCX 16 kb)

## Abbreviations

BMI: Body Mass Index
CSO: Chief Scientist Office
FFIT: Football Fans in Training
MRC: Medical Research Council
RCT: Randomised Controlled Trial
SDT: Self-Determination Theory
SPFL: Scottish Professional Football League
