# Supplementary material for: Men’s reactions to receiving objective feedback on their weight, BMI and other health risk indicators
Source: BMC Public Health. 2018 Feb 27;18:291. doi: 10.1186/s12889-018-5179-1 (PMC5830073; doi:10.1186/s12889-018-5179-1)
Supplement: Supplementary file 1 — Interview Topic Guide. (DOCX 16 kb) [file 12889_2018_5179_MOESM1_ESM.docx]

| **Additional file 1**  **Interview Topic Guide**  Before and after taking part in the FFIT programme you took part in measurement sessions at the football stadium or at home.     - I am very interested in hearing about your experiences of receiving feedback about these measurements; please tell me in as much detail as possible, what it was like having these measurements done and hearing any results?   Probe in relation to:   - Blood pressure measurement; - Body weight (BMI and waist circumference); - sedentary behaviour/physical activity questionnaire; - Completing self-report questionnaires; - Respiratory function - What was it like getting the feedback? Were there any things that pleased/surprised/upset you? - Did this feedback make you think differently about yourself?   - Prompt, in what ways did this information influence perceptions of health?   - Did any of the feedback make you want to make any changes in your life?   - If so, how did this information motivate any changes in behaviour?   - Have you discussed your feedback with anyone else? (Probe, with whom and what was discussed i.e. with family members, other men on FFIT programme etc) |
| --- |
